# Supplementary material for: Single Particle Automated Raman Trapping Analysis
Source: Nat Commun. 2018 Oct 15;9:4256. doi: 10.1038/s41467-018-06397-6 (PMC6189196; doi:10.1038/s41467-018-06397-6)
Supplement: Supplementary file 1 — Supplementary Information [file 41467_2018_6397_MOESM1_ESM.pdf]

SUPPLEMENTARY INFORMATION

Single Particle Automated Raman Trapping Analysis

Jelle Penders[1,2,3†], Isaac J. Pence[1,2,3†], Conor C. Horgan[1,2,3], Mads S. Bergholt[1,2,3], Christopher S. Wood[1,2,3], Adrian Najer[1,2,3], Ulrike Kauscher[1,2,3], Anika Nagelkerke[1,2,3] and Molly M. Stevens[1,2,3 *]

[1]*Department of Materials, Imperial College London, London SW7 2AZ, United Kingdom*

[2]*Department of Bioengineering, Imperial College London, London SW7 2AZ, United Kingdom*

[3]*Institute of Biomedical Engineering, Imperial College London, London SW7 2AZ, United Kingdom*

Correspondence: m.stevens@imperial.ac.uk

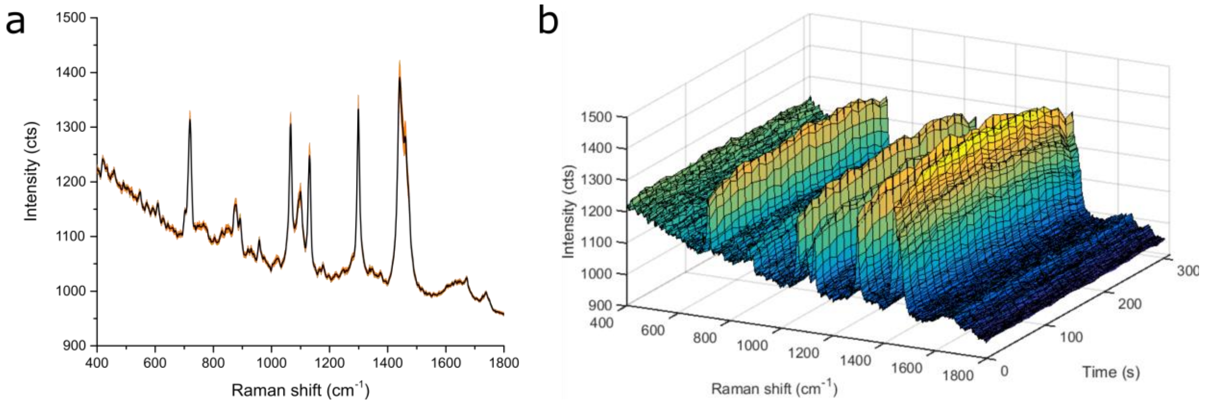

Supplementary Figure 1 | DPPC liposome photostability over time. DPPC liposome held in the optical trap for 5 minutes. (a) Averaged Raman spectra (mean ± s.d., n = 26), (b) surface plot of Raman spectra over time, showing minimal variation over time in the spectrum indicative of photostability.

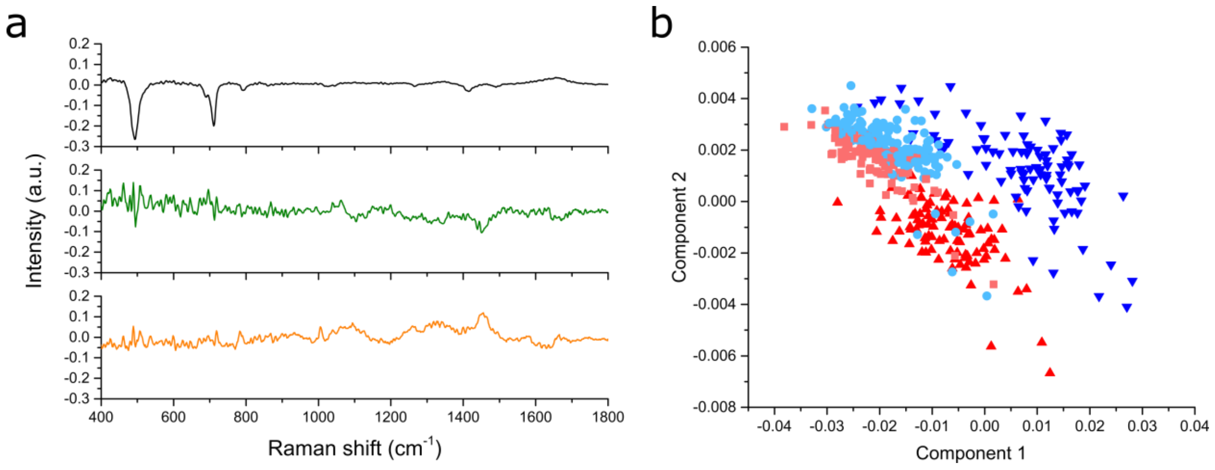

Supplementary Figure 2 | SPARTA polymersome composition analysis, PLSDA classification. 3 component PLSDA classification analysis of a 50-50 v/v % mixture of ABA and ABA-heparin polymersomes (n = 263). (a) PLSDA components 1, 2 and 3 (top to bottom), (b) PLSDA scores of component 1 and 2 and classification of ABA (light red) and ABA-heparin (light blue) like polymersomes, modelled using pure ABA (red, n = 99) and ABA-heparin (blue, n = 98) trapping runs.

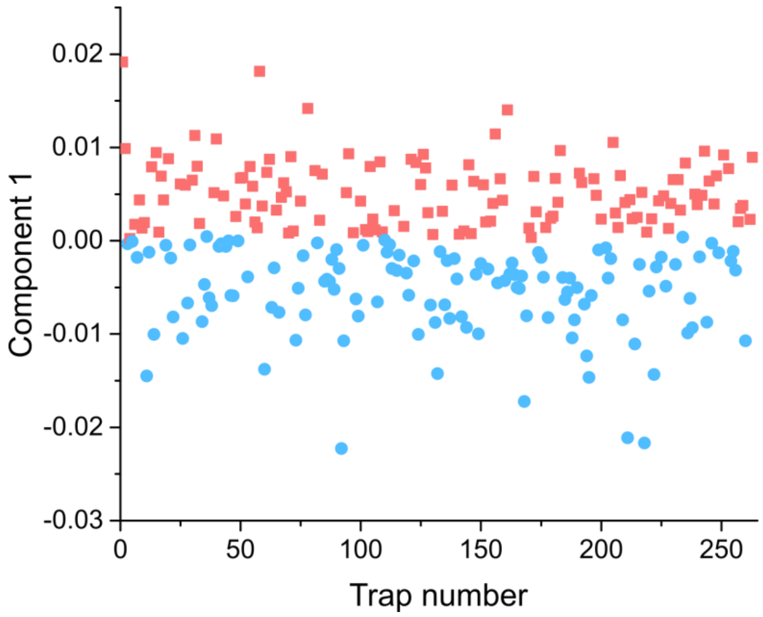

Supplementary Figure 3 | Classification versus trap number. PCA component 1 against the trap number of a 50-50 v/v % mixture of ABA and ABA-heparin polymersomes (n = 263), classified by PCA based Ward's clustering into ABA (red) and ABA-heparin (blue) populations. Plot shows a random distribution of trapping of both particles over time.

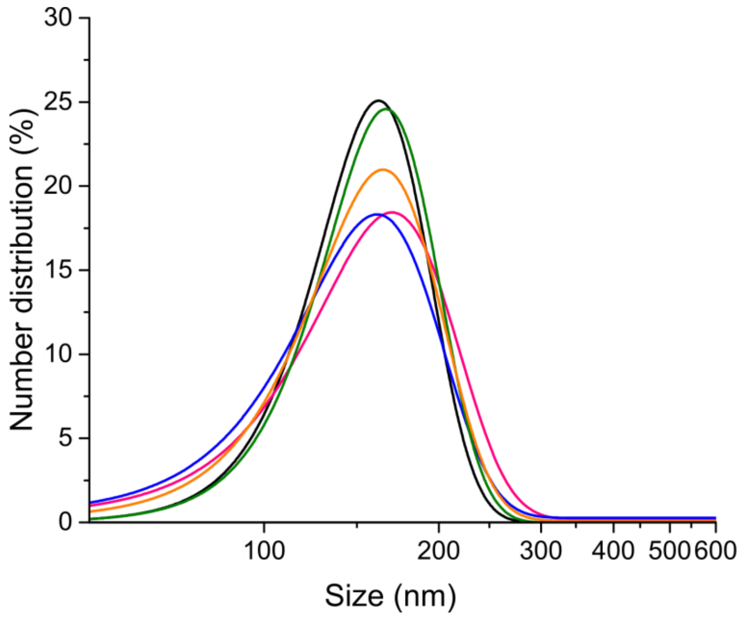

Supplementary Figure 4 | DLS number distributions of polystyrene particles during serial functionalisation. Gaussian fits of DLS number distributions for PS particles with sulfhydryl functionalisation (mean ± s.d.) (black, 157.5 ± 34.9 nm), disulphide and nitrobenzoic acid (red, 166.1 ± 46.6 nm) and disulphide and CYY functionalised particles (blue, 156.6 ± 43.5 nm), recovery of sulfhydryl after addition of TCEP to disulphide and nitrobenzoic acid functionalisation (orange, 162.1 ± 36.6 nm), and to CYY functionalisation (green, 160.3 ± 40.9 nm).

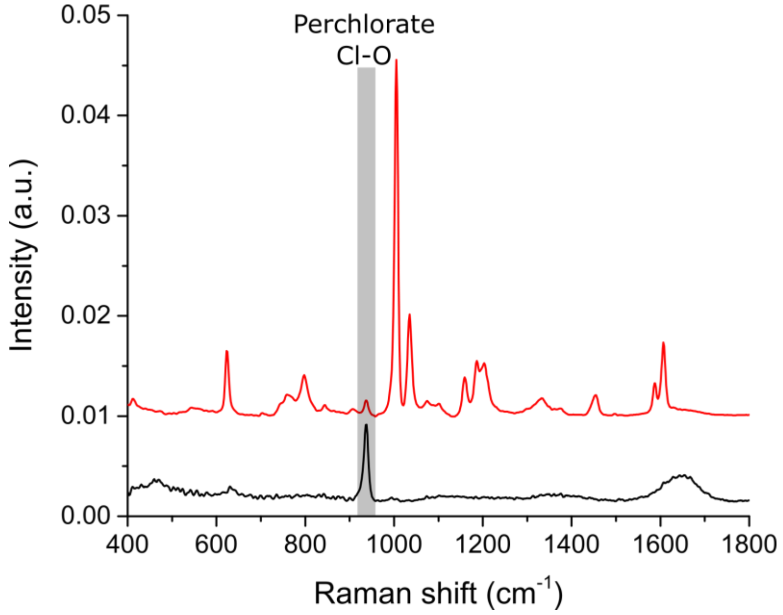

Supplementary Figure 5 | Perchlorate Raman spectra. Averaged Raman spectra of PBS (black, n = 234) and 200 nm polystyrene beads (red, n = 300), each supplemented with 50 mM sodium perchlorate. The perchlorate gives a single, clear solution marker peak at 938 cm^{-1}.

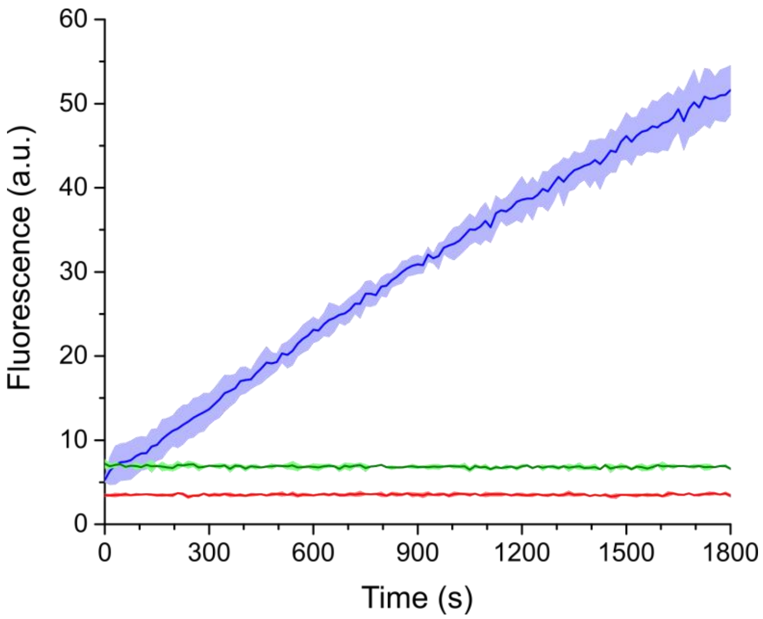

Supplementary Figure 6 | Fluorescence analysis of coumarin dye clicking. Copper catalysed click reaction of 3-Azido-7-hydroxycoumarin (Absorption/Emission = 404/477 nm) to 200 nm polystyrene particles functionalised with propargyl amine resulting in fluorescence when forming the triazole product, (n = 3, mean ± s.d.) clicking conditions (blue), no change in fluorescence in absence of dye (red) or absence of copper sulphate (green).

Supplementary Table 1 | Raman peak vibration assignment.

**Figure**	**Vibration**	**Raman shift (cm^{-1})**	**Reference**
3 a,b,c	*C-D* stretch	2105	1
3 d	*Si-O-Si* stretch	490	2
3 d	*Si-C* sym. stretch	709	2
3 d	*C-C* stretch glucose/saccharide	930	3
3 d	Glucose/saccharide	1070	3
4 b,c	*S-S* stretch	452, 512	4, 5
4 b,c	*S-H* bend	936	5
4 b,c	*C=C* ring breathing (Tyrosine)	840, 860	3
6 b,c,d	*C≡C* stretch (Alkyne)	2129	1
6 b,c,d	*N≡N* stretch (Azide)	2116	1
6 b,c,d	Triazole ring stretch	1331	3, 4
Supplementary Figure 2	*Cl-O* stretch (Perchlorate)	938	6

Supplementary References

1. Yamakoshi, H. et al. Alkyne-tag Raman imaging for visualization of mobile small molecules in live cells. *J. Am. Chem. Soc.* **134**, 20681-20689 (2012).
2. Cai, D., Neyer, A., Kuckuk, R. & Heise, H.M. Raman, mid-infrared, near-infrared and ultraviolet–visible spectroscopy of PDMS silicone rubber for characterization of polymer optical waveguide materials. *J. Mol. Struct.* **976**, 274-281 (2010).
3. Movasaghi, Z., Rehman, S. & Rehman, I.U. Raman spectroscopy of biological tissues. *Appl. Spectrosc. Rev.* **42**, 493-541 (2007).

4. Yoo, B.K. & Joo, S.-W. In situ Raman monitoring triazole formation from self-assembled monolayers of 1,4-diethynylbenzene on Ag and Au surfaces via “click” cyclization. *J. Colloid Interface Sci.* **311**, 491-496 (2007).
5. Bazylewski, P., Divigalpitiya, R. & Fanchini, G. In situ Raman spectroscopy distinguishes between reversible and irreversible thiol modifications in l-cysteine. *RSC Adv.* **7**, 2964-2970 (2017).
6. Cherney, D.P., Bridges, T.E. & Harris, J.M. Optical trapping of unilamellar phospholipid vesicles: investigation of the effect of optical forces on the lipid membrane shape by confocal-Raman microscopy. *Anal. Chem.* **76**, 4920-4928 (2004).
